# Supplementary material for: Design of Microbial Consortia Based on Arbuscular Mycorrhizal Fungi, Yeasts, and Bacteria to Improve the Biochemical, Nutritional, and Physiological Status of Strawberry Plants Growing under Water Deficits
Source: Plants (Basel). 2024 Jun 4;13(11):1556. doi: 10.3390/plants13111556 (PMC11175115; doi:10.3390/plants13111556)
Supplement: Supplementary file 1 [file plants-13-01556-s001.zip › plants-3018228-supplementary.pdf]

Table S1. Treatments evaluated in the interaction rhizobacteria-AMF-yeasts in strawberry plants grown under water deficit.

Treatments	Microorganism group								
	AMF			Yeasts			Rhizobacteria		
	F. mosseae	*C. lamellosum*	*C. claroideum*	*R. mucilaginosa*	*C. guillermondis*	*N. albida*	*P. frederiksbergensis*	*B. tequilensis*	*B. caledonica*
CS1	X			X			X		
CS2	X			X				X	
CS3	X			X					X
CS4	X				X		X		
CS5	X				X			X	
CS6	X				X				X
CS7	X					X	X		
CS8	X					X		X	
CS9	X					X			X
CS10		X		X			X		
CS11		X		X				X	
CS12		X		X					X
CS13		X			X		X		
CS14		X			X			X	
CS15		X			X				X
CS16		X				X	X		
CS17		X				X		X	
CS18		X				X			X
CS19			X	X			X		
CS20			X	X				X	
CS21			X	X					X
CS22			X		X		X		
CS23			X		X			X	
CS24			X		X				X
CS25			X			X	X		
CS26			X			X		X	
CS27			X			X			X
CS28	WS	WS	WS	WS	WS	WS	WS	WS	WS
CS29	WW	WW	WW	WW	WW	WW	WW	WW	WW

CS: consortium; WS: water-stressed control (30% water holding capacity (WHC)) uninoculated; WW: well-watered control (85% substrate WHC) uninoculated.

Table S2. Analytical parameters for spectrophotometric methods

Method	Standard	Equation	R^2	DL	QL	LR	CV (%)
Folin	Gallic acid	y=0.0007x+0.0523	0.994	36.3 mg L^{-1}	120.9 mg L^{-1}	120.9–500 mg L^{-1}	2.35
TEAC	Trolox	y=0.3785x+0.1253	0.992	0.11 mmol L^{-1}	0.38 mmol L^{-1}	0.38-0.7 mmol L^{-1}	12.09
CUPRAC	Trolox	y=2.5385x+0.0936	0.990	0.01 mmol L^{-1}	0.04 mmol L^{-1}	0.04-0.7 mmol L^{-1}	6.12
DPPH	Trolox	y=0.5861x+0.0167	0.991	0.09 mmol L^{-1}	0.30 mmol L^{-1}	0.30-0.7 mmol L^{-1}	5.79

DL: detection limit; QL: quantification limit; LR: linear range; CV: coefficient variation
